# Social learning from conspecifics and humans in dog puppies

**DOI:** 10.1038/s41598-018-27654-0

**Published:** 2018-07-05

**Authors:** Claudia Fugazza, Alexandra Moesta, Ákos Pogány, Ádám Miklósi

**Affiliations:** 1 Department of Ethology, Eötvös Loránd University, Budapest, Hungary; 2 WALTHAM Centre for Pet Nutrition, Freeby Lane, Waltham-on-the-Wolds, Melton Mowbray, UK

## Abstract

Social learning is especially advantageous for young individuals because it reduces the risks of trial-and-error learning, while providing an efficient way of acquiring information. Whereas adult dogs are known to excel in social learning skills, the ontogeny of this process has been mainly overlooked. The focus of our study was to investigate whether the capacity of social learning is already developed in dogs at an early age. We tested 8-week-old dog puppies on their ability to learn socially to open a puzzle box baited with food and on their capacity to retain the acquired information in their memory. Puppies were tested with conspecific and human demonstrators. We further investigated on whether demonstrations performed by their mother or by an unfamiliar conspecific model affected puppies’ learning trend differently. We found that social learning skills are present in 8 weeks old puppies and they remembered this experience for 1 hour. Puppies learned to solve the task from both conspecific and human demonstrators, thereby endorsing dogs’ flexibility in learning from different social partners. Unexpectedly, puppies were more likely to learn from unfamiliar conspecifics than from their mother, probably as a result of greater attention payed to the demonstration performed by the unfamiliar model.

## Introduction

Social learning is an efficient and fast mechanism to transfer information from more experienced (often, older) to less experienced (younger) individuals. Especially in species with prolonged parental care, such as some mammals and birds, where offspring have ample opportunities to learn various behaviours from the parents, social learning has a large impact on fitness by reducing the costs and dangers associated with individual learning^1,2^. Social learning has long been known to play a major role in information acquisition and cultural evolution in humans^3,4^. In non-human animals, most research into social learning so far, either took a functional approach by focusing on fitness consequences, or it investigated what kind of information is acquired and through what mechanisms^5^. Interestingly, more little research has been carried out in the context of ontogeny, i.e. individual development^6–8^.

Dogs evolved in the human environment, a milieu that is extremely complex, variable, rich in information to acquire and in models from which to acquire it. Dogs’ evolutionary background and development in human social groups makes them ideal subjects for investigations on social cognition as they readily and flexibly form social relationships, not only with conspecifics, but also with humans^9–11^. A number of studies showed that adult dogs can learn by observation in various contexts, both from conspecifics and from humans, and they can obtain different types of skills through social learning^12–14^. For example, Kubinyi *et al*.^15^ showed that, after observing a human releasing a ball by manipulating a lever mounted on a box, dogs are more successful in using the lever to achieve the same goal. For socially acquired information to be beneficial for the learner, it has to be remembered over time. Adult dogs are known to be able to remember and re-enact human actions after a delay of 1 hour and some of them even after delays up to 24 hours^16,17^ and training methods relying on social learning can enhance dogs’ memory and ability to generalize the trained actions^18^.

Surprisingly, while adult dogs’ ability to acquire information socially from conspecifics and from humans has been widely documented, the role of social learning during development has been overlooked so far. Only two studies have investigated social learning in dog puppies. Adler and Adler^19^ revealed that young puppies learn from a littermate to pull a small trolley, and Slabbert and Rasa^20^ reported that puppies that observed their mother’s drug detection training learned a similar task faster than puppies that were not provided such experience. Young animals have less experience with their environment and are more vulnerable than adults in general. Hence, young individuals are typically inclined to acquire information socially and early learning through observation can have a significant effect on the development of their skills^21^.

Vertical transmission of information (i.e., from parents to offspring) is thought to be one of the major pathways of social learning, on which cultural evolution largely depends^4,22^. Learning socially from the parents is widespread and well-documented in different species including - among others – dolphins^23,24^, chimpanzees^25^ and oystercatchers^26^.

While human infants acquire a large amount of information from their mother^27^, she is not always the model from which the child learns preferentially and this may vary in different contexts^28,29^. In other species, young individuals of group living animals have many opportunities to interact with various demonstrators other than the parents^30^, thus they may be flexible in acquiring information socially not only from their own parents (vertical transmission) but also from other non-kin individuals of former generations (oblique transmission) and from other different individuals of their social group (horizontal transmission)^31,32^. However, most animals rarely interact with unfamiliar models (i.e., individuals that are not members of their social group) and consequently chances of learning from those are scarce. Dogs differ in this regard, because the dog-human social environment is very heterogeneous and can change fast (e.g., dogs and owners often encounter and spend time with individuals that are not from their social group). These are conditions that are rarer in other species’ environments. The extreme plasticity of this social environment gives dogs the chance to interact with different social partners, including unfamiliar individuals. Thus, in the case of dogs, the ability to acquire information from unfamiliar models in social learning situations constitutes a valid and particularly interesting topic, from an ethological perspective.

We tested the ability of 8-week-old puppies to learn to solve a problem by observing conspecifics and humans in two conditions (learning from conspecifics condition and learning form human condition). The focus of our study was to investigate whether the ability of social learning is already developed in dogs at this early age and whether, like adult dogs^16,17^ they can retain in their memory the acquired information if tested again after 1-hour delay. In the human condition, as puppies of this age still live in the kennel, with only limited interactions with the breeder, we tested them with the Experimenter as the demonstrator. In the conspecific condition, we further investigated whether puppies learn with similar success from different demonstrators: their mother and an unfamiliar conspecific. Adult dogs are more likely to learn from humans if the human demonstrator makes eye contact with them and calls their attention using vocal signals^33^. Thus, we used such cues in the case of the human demonstrator. To control for social facilitation processes, in which the presence of another individual enhances the subject’s exploratory activity or motivational levels without the subjects acquiring information socially^34,35^, control puppies were exposed to the demonstrator (human or conspecific), eating food close to the apparatus, but not solving the problem.

Due to the variability and complexity of the social and asocial environment that dogs need to fit in when sharing their lives with humans, we hypothesised that the ability to learn socially develops early in dog puppies. Thus, we expected 8-week old puppies exposed to a model solving the problem to be more likely to solve it than non-exposed puppies. We also hypothesised that dog puppies, similarly to adults^13,36,37^, would show a remarkable flexibility in learning socially not only from their mother, but also from unfamiliar conspecific and heterospecific models that do not belong to their social group. Thus, we expected puppies exposed to unfamiliar adult dogs and humans to be more likely to solve the problem than puppies that had no opportunity to learn socially.

## Results

### Social learning from conspecifics

#### Success in problem solving

**Figure 1:**
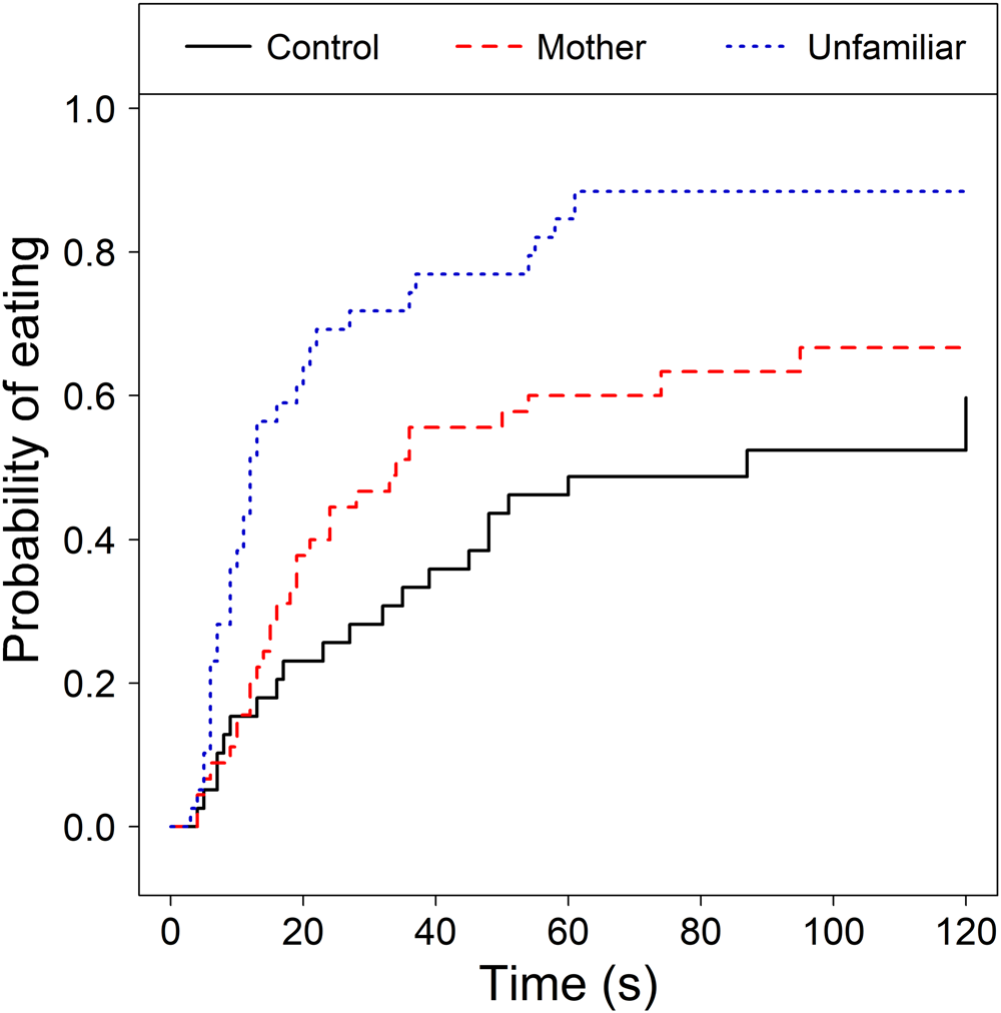
Effects of experimental group on successful performance (solving the problem and eating the reward). Puppies in the unfamiliar group - but not in the mother group – were more likely to solve the task compared to puppies in the control group.

The probability of problem solving (i.e. eating the food from the puzzle box) was significantly different between experimental groups (Cox Mixed Model of latency to eat, effect of experimental group: χ^2^_2_ = 8.84, P = 0.012), because puppies in the unfamiliar group were more likely to succeed than puppies in the control group (control → unfamiliar dog: Exp(β) = 5.80 [1.84; 18.31], z = 3.00, P = 0.003). Success of puppies in the mother group did not differ significantly from the control group (control → mother: Exp(β) = 1.78 [0.59; 5.40], z = 1.02, P = 0.310; Fig. 1). In addition, puppies had similar success in problem solving during the retention trial and the two preceding test trials (effect of trial: χ^2^_2_ = 0.36, P = 0.834).

#### Duration of looking at the demonstration

To assess whether higher success in the unfamiliar group was due to increased attention to the demonstration by the unfamiliar dog as opposed to by the mother, we compared the proportion of demonstration time spent by the puppies looking in the direction of the demonstration in these two groups. In the mother group, duration of demonstrations was 85.5 ± 25.2 sec (mean ± SD), of which puppies looked at the demonstrations for 30.5 ± 11.8 sec (36.3% ± 13.4% of the demonstration time). In the unfamiliar group, duration of demonstrations was similar: 82.1 ± 23.5 sec. However, puppies spent more of this time looking at the demonstration: 44 ± 14.6 sec (57% ± 20.3% of the demonstration time), and this difference was significant (t_24_ = 3.14; P = 0.004).

#### Action used to solve the task

While the action used by the demonstrators varied from dog to dog (N = 8 subjects used their muzzle to open the puzzle box, N = 3 subjects used their paw and N = 3 subject varied from test trial to test trial between using either paw or muzzle), all the puppies (except one subject in its second trial) invariably used their muzzle to solve the task. The subject that used its paw in one trial had observed its mother solving the problem by using its paw.

### Social learning from humans

#### Success in problem solving

**Figure 2:**
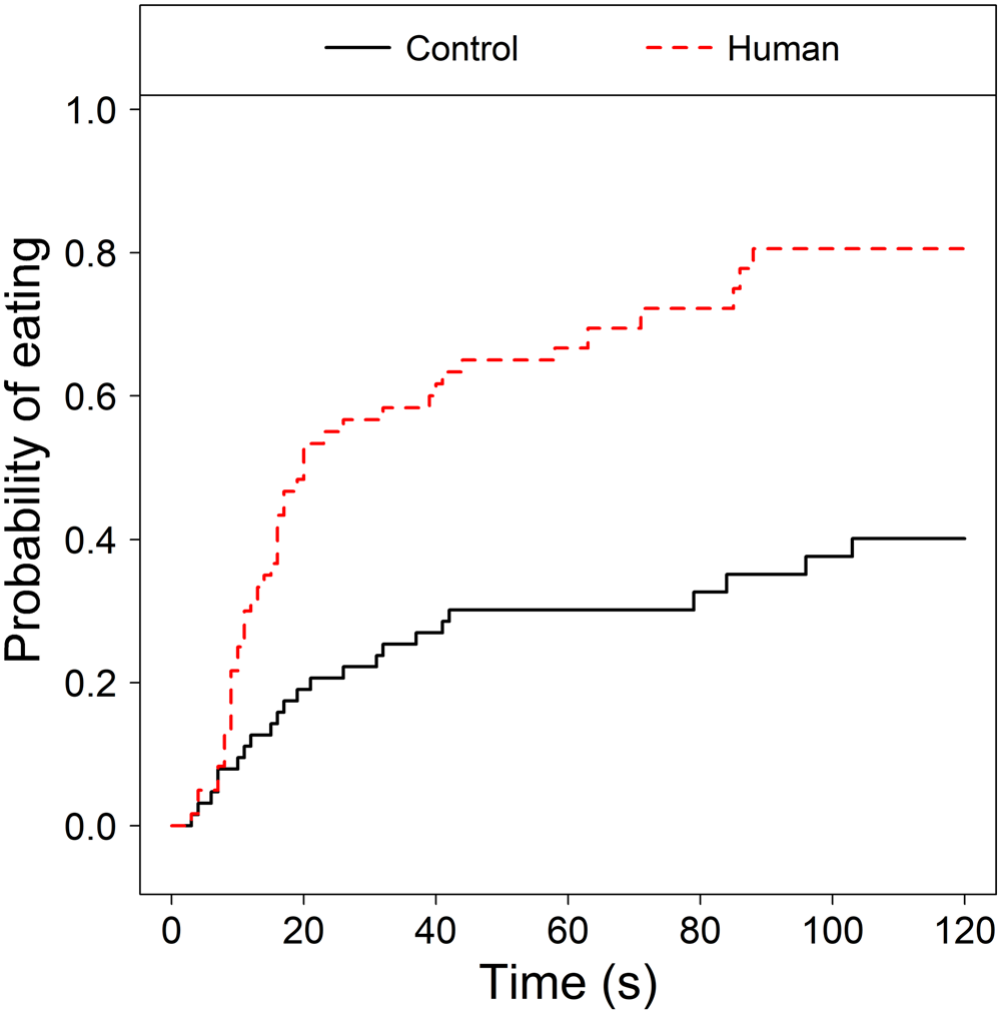
Effects of experimental group on successful performance (solving the problem and eating the reward). Puppies observing a human demonstration were more likely to succeed in opening the puzzle box than those in the control group.

The probability of problem solving was significantly different between experimental groups (Cox Mixed Model of latency to eat, effect of experimental group: χ^2^_1_ = 11.63, P < 0.001), as puppies in the human demonstration group were more likely to succeed than puppies in the human social facilitation group (control → human: Exp(β) = 4.24 [1.84; 9.77]; z = 3.39; P < 0.001; Fig. 2).

**Figure 3:**
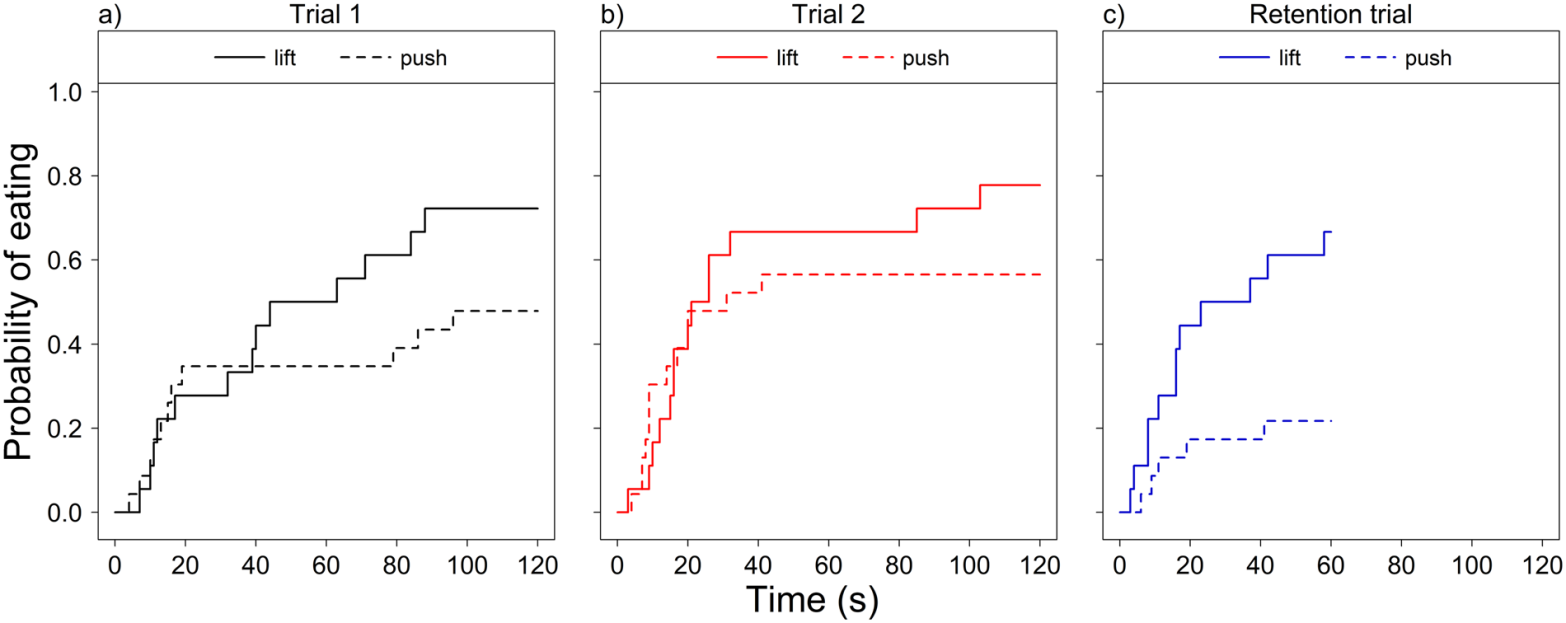
Test trial-specific effects of type of task on successful performance (solving the problem and eating the reward) of dog puppies in the human condition. Subjects in the 1 hour retention trial (trial 3) succeeded more likely with the lift lid task than with the push lid task compared to trial 1; Fig. 3a–c) illustrates the effect of type of task in trial 1, trial 2 and retention trial, respectively.

The type of task also had a test trial-specific effect on the subjects’ performance (Cox Mixed Model of latency to eat, effect of trial x task interaction: χ^2^_2_ = 7.37, P = 0.025), because puppies in the retention trial (trial 3) were more likely to succeed with the lift lid task than with the push lid task compared to trial 1 (lift lid → push lid in trial 3 vs. trial 1: Exp(β) = 0.25 [0.06; 1.05]; z = −1.89; P = 0.058; Fig. 3).

## Discussion

This study provides experimental evidence that dogs as young as 8 weeks old have already developed some social learning skills. Puppies observing the demonstration performed by an unfamiliar dog - but not those observing their mother - were more likely to succeed in manipulating a puzzle box and obtaining the reward than puppies in the social facilitation group. Puppies observing the experimenter solving the task were also more likely to succeed than puppies in the human social facilitation group. These results indicate that social learning skills develop early in the ontogeny of dogs, as 8-week-old dog pups are already able to acquire information socially about some aspects of the environment, specifically, about how to solve a task to obtain food or which objects to interact with. Moreover, puppies that were exposed to the demonstration in the first two trials performed still better than control puppies even after a 1-hour delay, implying that some information was acquired and retained in their memory.

As expected, puppies did not learn only from a conspecific, but also from a demonstrator of a different species. This finding is consistent with the body of knowledge from studies on adult dogs’ ability to acquire information socially from humans and shows that dogs’ flexibility, enabling them to learn from heterospecific partners, develops early in ontogeny. Pongrácz *et al*.^13^ found that adult dogs acquired a detour behaviour to obtain a reward when it was shown by human demonstrators. Although dogs had difficulties with detouring the fence on their own, they could learn this response quickly from both unfamiliar human^13,36^ and dog demonstrators^37^. Moreover, similar to the 8-week-old subjects of our study, adult dogs were shown to be able to learn from humans to manipulate an object to obtain a reward^15^.

An unexpected result of our study is that puppies observing an unfamiliar dog demonstrator were more likely to solve the task than control puppies, whereas those observing their mother were not. We suggest multiple, mutually non-exclusive explanations for this result. One possible explanation is that puppies paid more attention to the unfamiliar dog demonstrator than to the mother. Indeed, we found that puppies looked longer at the demonstrations performed by the unfamiliar dog than at those performed by their mother. There are two possible explanations for this biased attention. First, the unfamiliar dog was a novel stimulus for the puppies and might have captured their attention more than their mother. Puppies, therefore, spent more time looking at the unfamiliar dog and, as a consequence, they had greater opportunity to learn from its demonstrations. Similarly, rats were shown to prefer information from novel individuals, probably as a result of the greater amount of time spent by observers in proximity of them and sniffing them^38^. Novelty seems to stimulate more interest with respect to social information also in frugivorous bats as they seem to prefer information from novel social partners^39^. Second, it is possible that puppies use different strategies to obtain food from their mother and from unfamiliar adults, based on their possible interactions with them. In our experiment, the observer puppies were physically separated from the demonstrators, however, this is unusual in natural settings, where typically demonstrator and observer are together^40^. It has been noted that, when scrounging is a possibility, it prevents social learning to emerge^41,42^. Puppies can often ‘scrounge’ from their mother, who shares food with them and may even regurgitate it for the purpose of feeding her offspring^43^. On the contrary, it is unlikely that unfamiliar dogs would allow puppies to steal their possessions. We argue, therefore, that social learning might not be an optimal strategy to obtain food from the mother, while learning by observation might be the only possibility in the case of interactions with an unfamiliar dog. Furthermore, we note that in human infants, the tendency to learn from the mother or from unfamiliar models seems to be context dependent so that in some situations the familiarity of the model (i.e., the mother) does not favour social learning^29,44,45^. For example, toddlers can learn a novel action by observing a completely unfamiliar model, when s/he does not address them directly^30^. Thus, we do not exclude that in a different context, puppies may learn from the mother as well.

Our finding of puppies in the unfamiliar group – but not puppies in the mother group - learning more likely than control puppies is in contrast with results obtained in other species. Kittens learn to press a lever for food more rapidly from their mother than from an unfamiliar adult^46^. Although domestic cats show a certain level of sociality^47^, they evolved from a non-gregarious ancestor, whose kittens are rarely exposed to familiar demonstrators different from the mother. On the contrary, young individuals of group living, gregarious animals that remain in their social group for a prolonged period have ample opportunities to observe other familiar demonstrators of their social group^31^. Dogs evolved from a gregarious species, whose offspring is likely to be exposed to various models within the social group since a young age. Wolf pups emerge from the den and are exposed to the other members of the pack from around the age of 3 weeks^48,49^. Similarly, dog puppies begin to explore the environment from around 3 weeks of age^50^, thus having the opportunity of learning from other group members.

Our subjects were tested with unfamiliar models not belonging to the social group. Most animals of group-living species spend no or very little time with non-group members. Therefore, if social learning takes place, it likely involves observing group members, rather than unfamiliar models. Companion dogs are special in this respect, because they often leave the ‘home pack’, and meet with unfamiliar dogs and humans. Their ability to cope with the extreme plasticity of this social environment may also include the tendency to pay attention to unfamiliar social stimuli and take advantage from learning from them. For adult dogs, owners and strangers were found to be equally effective as demonstrators in a detour task^13,51^. Thus, it is possible that, during their development, dogs learn to attend both their mother and unfamiliar demonstrators because both can provide useful information. Moreover, and in line with human studies^29,30^, we do not exclude that the tendency to pay attention to and learn from different models might be context and/or task dependent in young dogs too. To further investigate on the role of familiarity, future research could investigate on puppies’ tendency to learn socially form familiar models other than their mother.

The results obtained in the retention trials provide insights on dog puppies’ memory of socially acquired information. Puppies could retain information in their memory for a 1 hour delay. Our experimental design, however, does not allow separating the effect of the observation of the demonstration from that of the motor practice performed during the first two trials. Thus, the subject’s memory in the retention trial may rely on both the socially acquired information and the individual experience. In the conspecific condition, trial did not have any significant effect on the puppies’ success, i.e. their performance did not differ in the retention trial compared to the first two trials with demonstration. Thus, puppies acquired a skill during exposure to the demonstration (and possibly also when performing the task) that was retained in their memory for at least 1 hour. This is consistent with previous findings on adult dogs’ memory of socially acquired information, showing that they typically remember others’ actions for such retention intervals^16,17^. In the human condition, puppies were more successful in lifting the lid than pushing the lid in the retention trial compared to the first two test trials. This suggests that their memory of the acquired information varies with the specific task (and with specific demonstrators), and some tasks might be more difficult to encode and remember than others. This cautions against generalizing findings obtained with a single specific task, because memory of the acquired information might be task-dependent.

With regard to the action used to solve the task, we did not find any variation in the motor action used by the puppies, which would have allowed to test for imitative learning^52^. While dog demonstrators varied between using their paw or muzzle, all puppies used their muzzle to open the puzzle box. This may suggest that imitative abilities develop at a later time in ontogeny. However, other possible explanations may account for this result. Our tasks required some precision in paw movement to allow the opening of the box. Thus, the invariable use of muzzle actions by the puppies may also indicate that, by this age, their motor skills in manipulative actions requiring precision with their paws is not yet completely developed, making it more likely that they would use muzzle, rather than paws, for manipulating objects. For example, developmental constrains are also known to affect foraging behaviour in juvenile birds^53,54^. In addition, we cannot exclude the possibility that it was easier for puppies to learn by other social learning processes (e.g., goal emulation^55^ and/or stimulus enhancement^56^), thus not allowing imitation to emerge in our experimental setup.

In conclusion, social learning skills develop early in dogs’ ontogeny. 8-week-old puppies are already able to acquire socially information on how to manipulate an object to obtain a reward and they can retain this information in their memory. They learn not only from experienced conspecific models, but also from humans, thereby endorsing dogs’ remarkable flexibility in interacting with heterospecifics as social partners. Puppies pay more attention to an unfamiliar conspecific demonstrator than to their mother and, as a consequence, there are more likely to learn from this unfamiliar model. Future studies may further investigate on the ontogeny of social learning skills in dogs, by testing subjects in different stages of development and on their retention of socially acquired information, by disentangling the role of observation from motor practice. Moreover, future studies may focus on the role of familiarity of the model in social learning situations.

## Methods

The puppies were tested in two conditions in which they observed either skilful adult dogs (their mother or an unfamiliar dog; condition 1) or a human (condition 2) manipulating an object (puzzle box) to obtain food. Then the subjects were allowed to interact with the same object. All subjects were tested in both conditions. In each one, puppies of the same litter were allocated to different experimental groups. In both conditions, we included a social facilitation control group exposed to the demonstrator not solving the problem. Allocation to the groups (experimental or social facilitation control) was counterbalanced between test conditions (mother/unfamiliar dog or human), so that puppies that were in the control group in one condition were allocated to experimental group in the other condition. Therefore, all puppies observed the demonstration in at least one condition (Table 1).

**Table 1:** Scheme of the experimental design.

**HUMAN CONDITION**							
**Litter #1**				**Litter #2**			
Control group		Human group		Control group		Human group	
Push lid task				Lift lid task			
**CONSPECIFIC CONDITION**							
**Litter #1**				**Litter #2**			
Mother group	Unfamiliar group		Control group	Mother group	Unfamiliar group		Control group
Lift lid task				Push lid task			

All puppies were tested in two conditions in randomized order: human and conspecific conditions. Within every condition, littermates were semi-randomly allocated to different experimental groups. Subjects were tested on two tasks, counterbalanced across conditions.

### Subjects

The tests were conducted in Italy and Hungary between August 2016 and March 2017. We enrolled 48 dog puppies originating from 8 litters of various breeds (Swiss hound N = 3 puppies, Border Collie N = 5, Shetland sheep dog N = 4, Belgian Tervueren N = 6, Mudi N = 8, Labrador Mix N = 8, Schnauzer mini N = 7 and a mixed breed litter with N = 7 puppies). The 7 Schnauzer puppies, however, later had to be excluded from the experiment due to lack of motivation for food, resulting in 41 puppies from 7 litters in our analyses. Puppies were tested at 8 weeks of age, before adoption, while still living with their mothers at the breeders’ facilities. Puppies were kept with their mother, in kennels or rooms in the breeder’s facilities. The puppies received daily interactions with the breeders taking care of them (e.g., cleaning the kennels and feeding).

### Experimental setup

**Figure 4:**
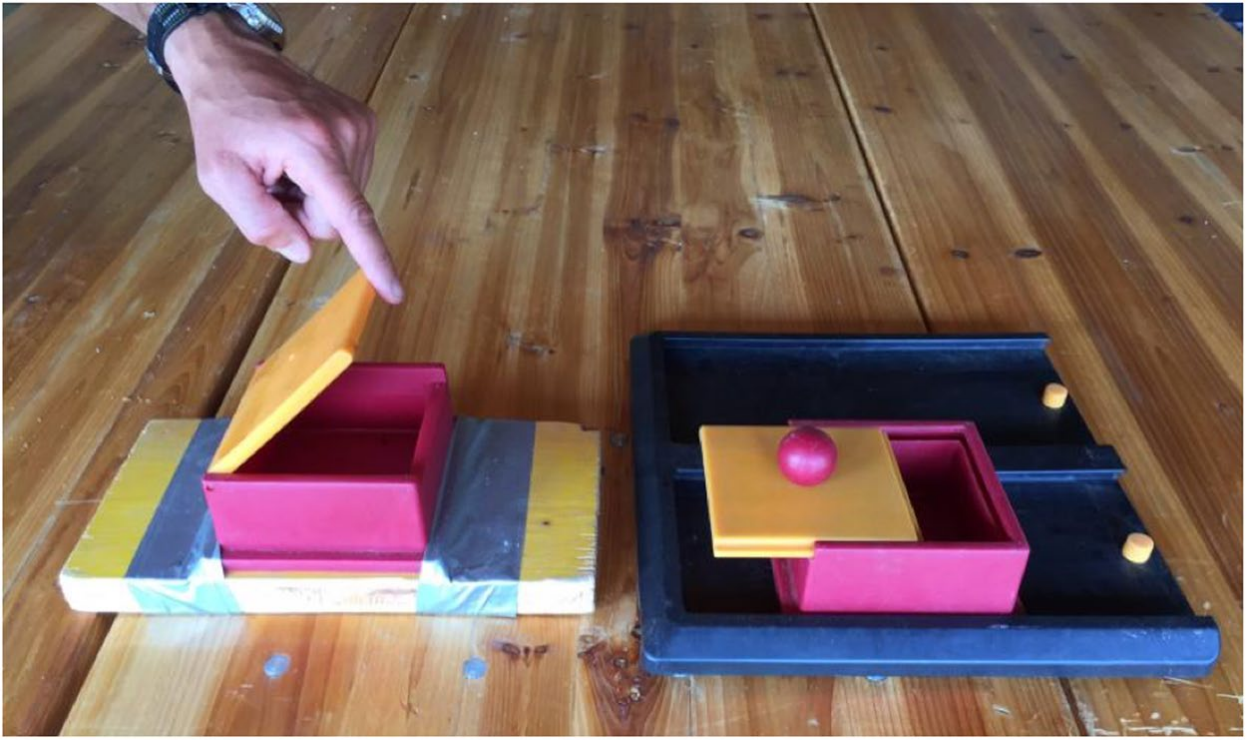
The two types of puzzle boxes (Trixie, Poker box) used for different tasks in the tests (lift lid on the left; push lid on the right).

We used two puzzle boxes presenting two problem solving tasks (from “Poker box” produced by Trixie):

- Lift lid: to open the box and obtain the food, the subject had to pull up the lid (Fig. 4, left panel);

- Push lid: to open the box and obtain the food, the subject had to push the lid horizontally, making it slide to a side (Fig. 4, right panel).

The two plastic boxes (cm 13 × 13 × 5.5) were mounted on two different bases, as to stabilise the object (Fig. 4).

Allocation of the tasks to condition 1 or 2 was counterbalanced - i.e., if litter 1 was tested on task ‘lift lid’ in the dog condition and on task ‘push lid’ in the human condition, then litter 2 was tested on task ‘push lid’ in the conspecific condition and on task ‘lift lid’ in the human condition; Order of administration of the two conditions and tasks were randomized.

The tests were performed at the breeder’s facility or home, in an area familiar to the puppies. We delimitated the testing area with a puppy fence (3.5 × 3.5 m). During the demonstrations, the subject was placed in a crate (1 × 0.4 m) from which it could watch the demonstrations. The crate was placed inside the puppy fence, at 1.5 m from the apparatus.

### Condition 1: Learning from conspecifics

#### Preliminary training

We trained two dogs as demonstrators for every litter: the mother of the puppies and an adult dog owned by the breeder but unfamiliar to the puppies. The demonstrator dogs were trained by operant conditioning methods until they performed the given task reliably (i.e. opening the puzzle box and eating the reward); we considered the training successful and completed it when the demonstrator dog was able to perform it successfully 6 times in a row. All demonstrator dogs could be trained within a short (max. 15 minutes) training session. The dogs were allowed to open the puzzle box with their spontaneous type of action (by pushing/pulling the lid with paw or muzzle).

#### Warming up

Before testing, all puppies (i.e. siblings from the litter) spent 10 minutes together in the testing area to ensure that they were comfortable there. To ensure that the puppies were motivated to eat the food we used during the test (sausages or dog treats), some pieces were given to them and in the tests, we used the type of food that they would eat immediately.

When the puppies were removed from the testing area, the demonstrator dog was introduced and was allowed to perform the predetermined task 3 times before the tests started. This was done to ensure successful demonstrations during the test.

#### Test procedure

1. The experimenter placed the apparatus (puzzle box) in the centre of the testing area;
2. The experimenter carried the puppy to the testing area in his/her hands and placed it close to the opened puzzle box, from which the puppy was allowed to eat 4 pieces of food, one-by-one. This was done to provide all the subjects with experience about presence of food in the puzzle box and to increase the probability they would be motivated to pay attention to the demonstration during the test;
3. The breeder (or a helper when the breeder was not available) placed the puppy in the crate and brought the demonstrator dog in the testing area. The demonstrator dog was then held then on leash by the experimenter close to the apparatus;
4. Before every demonstration, the experimenter attracted the puppy’s attention by smacking her lips and calling the puppy with a gentle high-pitched voice until the puppy’s head was oriented towards the demonstration area;
5. The demonstrator dog was held on leash and allowed to access the puzzle box to perform the demonstration (see below for description of the differences between the experimental groups);
6. The demonstrations were repeated 6 times;
7. After every demonstration the breeder (or helper) obstructed the puppy’s view placing a panel in front of the crate, while the experimenter refilled the apparatus with food;
8. After the 6^th^ demonstration, the experimenter led the demonstrator away from the testing area;
9. The puppy was released from the crate by opening its door. If it did not exit immediately, it was carried by the experimenter right out of the door;
10. The puppy was let free in the testing area (*test trial*). The trial was terminated either when the puppy solved the task (opening the box and eating the food) or after two minutes (whichever was reached first);
11. This procedure (i.e., 6 demonstrations and a problem solving test trial) was repeated twice;
12. To test whether puppies remembered solving the task, after 1 hour delay all subjects were re-tested with the puzzle box for 1 minute, but this time without demonstration (*retention trial*). Time of the retention trial was reduced to one minute to minimize the chance for trial and error learning.

#### Experimental groups

Puppies of the same litter were randomly allocated into 3 experimental groups:

#### Mother and unfamiliar dog demonstrators

In the mother (N = 15 puppies) and unfamiliar groups (N = 13 puppies), the demonstrator dog was held on leash by the experimenter and was allowed to solve the task 6 times in a row.

#### Dog social facilitation control (N = 13)

The same procedure was used for the control puppies (N = 13), which were exposed to the mother dog while she was eating the same amount of food close to the puzzle box, but without displaying solving the task (i.e., the pieces of food were delivered one by one by the experimenter).

### Condition 2: Learning from humans

Puppies were tested for their ability to learn from a human demonstrator (CF). In condition 2, puppies were required to solve the other task - i.e. the one that was not used in condition 1 for the given litter. Between the two conditions an interval of minimum 1 hour, maximum 2 hours elapsed.

Before performing the demonstration, the human demonstrator attracted the subject’s attention towards herself and then, differently from the conspecific condition, she kept the communication with the subject throughout the demonstration by alternating her gaze between the subject and the apparatus, in order to ensure the subject would pay attention to the whole demonstration^33^.

The procedure was similar to the one described above for condition 1, but the puppies were divided into 2 experimental groups:

*Human* (N = 20): puppies were exposed to the experimenter solving the task by opening the puzzle box by lifting or pushing the lid with her hand and eating a piece of food from the box;

*Human social facilitation control* (N = 21): puppies were exposed to the experimenter eating the same amount of food pieces, one by one, close to the apparatus, but not solving the task.

Apart from the brief interactions immediately before the experiment (i.e., carrying the puppies to the fenced area during the warming up and delivering some food to assess their motivation), the experimenter had no pre-test interaction with the puppies.

### Data collection and analysis

All tests were video recorded for later analysis. From the videos, we measured the subjects’ latency to success in solving the task (i.e., time in seconds, measured from when the subjects were released from the crate, until they opened the puzzle box and inserted their muzzle into it to take the food reward). In addition, we aimed at investigating the possible explanation for different success of subjects observing the unfamiliar dog or the mother demonstrators. Thus, in the ‘learning from conspecific’ condition, we also measured the total time of demonstrations (i.e., time needed by the model dogs to solve the task) and the time spent by the puppies looking at it (i.e., the puppy’s head was oriented towards the direction of the demonstrator).

Due to the methodological differences between the demonstrations in the two conditions (i.e., the human demonstrator - but not the dog - performed it with gaze alternation), we did not compare data between conditions, but only between experimental groups within condition.

We used the R statistical environment (v. 3.2.3; R Development Core Team, 2017) to analyse our data. Latencies (seconds) to solving the problem (i.e., eating the food from the puzzle box) were analysed in separate Cox Mixed Models^57^ with occurrence of eating as terminal event. Puppies that did not solve the problem were treated as censored observations. All initial Cox Mixed Models included experimental group, task and test trial (factor with three levels: 1^st^ trial, 2^nd^ trial and retention trial) as fixed effects and subject nested in litter as random term. Stepwise model selection was based on AIC values, and the effects of explanatory variables were analysed by likelihood ratio tests: we provide χ^2^ and p values of likelihood ratio tests of models with and without the explanatory variable. Hazard ratios (Exp[β]) with 95% CI between levels of a given fixed effect are also given.

To investigate whether attentional factors affected the success of puppies in solving the task with the different conspecific demonstrators, we compared the proportion of demonstration time spent by the puppies looking in the direction of the demonstration between experimental groups with unfamiliar dog and mother as demonstrators with unpaired *t* test. This analysis was restricted to include only those test trials in which the head of the puppy was always visible from the camera during the demonstrations (14 subjects in the mother group and 12 subjects in the unfamiliar group).

We also observed and intended to compare the action (i.e. body part - paw or muzzle) used by the demonstrator dogs and by the subjects for opening the puzzle box. All puppies, however, used their muzzle to solve the tasks, thus we did not analyse this data statistically.

### Data availability

The datasets generated and analysed during the current study are available from the corresponding author on reasonable request.

### Ethical statement

The authors confirm that the experiments reported in this paper are in accordance with the current Hungarian laws in regard to animal protection. The experimental protocol was approved by the ethical committee (AWERB) at Waltham Centre for Pet Nutrition on 2^nd^ November 2015.
